# YKL‐40 as a Biomarker of Inflammation in Chronic Insomnia: A Potential Pathway to Alzheimer's Disease

**DOI:** 10.1002/alz70858_098789

**Published:** 2025-12-25

**Authors:** Rayan Daoudi, Marie‐Josée Quinn, Julie Otis, Caroline d'Aragon, Alex Desautels, Mélanie Vendette, Erlan Sanchez, Julie Carrier, Nadia Gosselin, Rébecca Robillard, Béatriz Oliveira, Nicole Lazarovici, Andrée‐Ann Baril

**Affiliations:** ^1^ Center for Advanced Research in Sleep Medicine, Hôpital du Sacré‐Coeur de Montréal, CIUSSS‐NIM, Montréal, QC, Canada; ^2^ Université de Montréal, Montréal, QC, Canada; ^3^ Centre Intégré de Traumatologie, Hôpital du Sacré‐Coeur de Montréal, CIUSSS‐NIM, Montreal, QC, Canada; ^4^ University of Ottawa, Ottawa, ON, Canada; ^5^ Mcgill University, Montréal, QC, Canada; ^6^ Center for Advanced Research in Sleep Medicine, Hôpital du Sacré‐Coeur de Montréal, CIUSSS‐NIM, Montreal, QC, Canada; ^7^ Université de Montréal, Montreal, QC, Canada

## Abstract

**Introduction:**

Some sleep disturbances and disorders have been suggested as pro‐inflammatory conditions acting as risk factors for Alzheimer's disease (AD). YKL‐40, a protein secreted by astrocytes and microglia during neuroinflammation has been documented as a promising biomarker for neuroinflammatory processes and AD risk, and may be elevated in the context of sleep disorders, but this hasn’t yet been investigated. This study aimed to characterize the relationship between sleep—defined as insomnia, its severity, and various sleep disturbances—and plasma levels of YKL‐40, while also exploring the influence of potential confounders. These findings could provide insight into how sleep may act as modifiable risk factors for AD.

**Materials and Methods:**

The study included 40 participants with clinically diagnosed insomnia (61.18 ± 8.32 years, 25W) and 34 controls (64.44 ± 5.86 years, 12W). Plasma YKL‐40 concentrations were measured by ELISA. Sleep metrics were obtained through full‐night polysomnographic recordings. ANCOVAs were conducted to compare YKL‐40 concentrations between groups (1) insomnia vs. controls (2) non‐severe insomnia vs. severe insomnia. Secondly, linear regressions were used to analyze the relationship between Insomnia Severity Index (ISI) scores and sleep characteristics with YKL‐40 levels.

**Results and Discussion:**

In the full sample (*n* = 74), no association was found between insomnia severity, insomnia status, nor any objective sleep characteristics and YKL‐40 concentration.

Among the participants diagnosed with insomnia, those with severe insomnia (ISI≥22, *n* = 12) exhibited higher YKL‐40 levels compared to participants with non‐severe insomnia (ISI<22, *n* = 28), and higher ISI scores were associated with higher YKL‐40 levels. These findings remained significant after adjusting for age, sex, evidence of other sleep disorders, proinflammatory conditions and behaviors and medication usage. However, they were no longer significant when adjusting for mental health symptoms measured with the Beck Depression Inventory and Beck Anxiety Inventory.

**Conclusion:**

Our findings suggest that insomnia severity in individuals diagnosed with insomnia is linked to elevated YKL‐40 concentrations, independently of multiple confounders. However, the comorbidity between insomnia and mental disorders may play a key role in the selective vulnerability to AD. Future studies should evaluate the interaction of insomnia severity and mental health symptomatology when predicting AD risk and related processes.

